# A review of lesbian, gay, bisexual, trans and intersex (LGBTI) health and healthcare inequalities

**DOI:** 10.1093/eurpub/cky226

**Published:** 2018-10-31

**Authors:** Laetitia Zeeman, Nigel Sherriff, Kath Browne, Nick McGlynn, Massimo Mirandola, Lorenzo Gios, Ruth Davis, Juliette Sanchez-Lambert, Sophie Aujean, Nuno Pinto, Francesco Farinella, Valeria Donisi, Marta Niedźwiedzka-Stadnik, Magdalena Rosińska, Anne Pierson, Francesco Amaddeo, Health4LGBTI Network, Rafik Taibjee, Igor Toskin, Kai Jonas, Dennis van Der Veur, Odhrán Allen, Thierry Troussier, Petra De Sutter

**Affiliations:** 1 School of Health Sciences, University of Brighton, Brighton, UK; 2 Centre for Transforming Sexuality and Gender, University of Brighton, Brighton, UK; 3 Department of Geography, Maynooth University, Maynooth, Ireland; 4 School of Environment and Technology, University of Brighton, Brighton, UK; 5 Infectious Diseases Section, Department of Diagnostics and Public Health, University of Verona, Verona, Italy; 6 CReMPE—Regional Coordination Centre for European Project Management, Veneto Region—Department of Health, The Verona University Hospital, Verona, Italy; 7 European Parliament’s Intergroup on LGBTI Rights, Brussels, Belgium; 8 ILGA-Europe, Brussels, Belgium; 9 Department of Neuroscience, Biomedicine and Movement, University of Verona, Verona, Italy; 10 Institute of Public Health – National Institute of Hygiene, Warsaw, Poland; 11 EuroHealthNet, Brussels, Belgium

## Abstract

**Background:**

Lesbian, gay, bisexual, trans and intersex (LGBTI) people experience significant health inequalities. Located within a European Commission funded pilot project, this paper presents a review of the health inequalities faced by LGBTI people and the barriers health professionals encounter when providing care.

**Methods:**

A narrative synthesis of 57 papers including systematic reviews, narrative reviews, meta-analyses and primary research. Literature was searched in Cochrane, Campbell Collaboration, Web of Science, CINAHL, PsychINFO and Medline. The review was undertaken to promote understanding of the causes and range of inequalities, as well as how to reduce inequalities.

**Results:**

LGBTI people are more likely to experience health inequalities due to heteronormativity or heterosexism, minority stress, experiences of victimization and discrimination, compounded by stigma. Inequalities pertaining to LGBTI health(care) vary depending on gender, age, income and disability as well as between LGBTI groupings. Gaps in the literature remain around how these factors intersect to influence health, with further large-scale research needed particularly regarding trans and intersex people.

**Conclusion:**

Health inequalities can be addressed via changes in policy, research and in practice through health services that accommodate the needs of LGBTI people. With improved training to address gaps in their knowledge of LGBTI health and healthcare, health professionals should work in collaboration with LGBTI people to address a range of barriers that prevent access to care. Through structural change combined with increased knowledge and understanding, services can potentially become more inclusive and equally accessible to all.

## Introduction

International research increasingly demonstrates that lesbian, gay, bisexual, trans and intersex (LGBTI) people are frequently marginalized and experience significant health inequalities.^1–6^ Reducing health inequalities is a fundamental goal of public health and is regarded by the European Union (EU) as being one of the most important public health challenges facing its Member States.^7–9^ This emphasis is vital as inequalities impact on both the health outcomes of LGBTI people as well as their experiences of accessing healthcare.^10^ Evidence suggests that LGBTI people are more likely than the general population to report unfavourable experiences of healthcare including poor communication from health professionals and dissatisfaction with treatment and care received.^11–13^ LGBTI patients can face bias and discrimination in healthcare settings,^13^^,^^14^ with trans patients reporting most dissatisfaction resulting in some avoiding medical treatment, including emergency care.^15^

Major legislative reform in recent years have resulted in significant progress towards achieving equality for LGBT people.^6^ Acknowledgement of the need to endorse and exercise the rights of LGBTI people are increasing within the EU where people are broadly protected against discrimination on grounds of sexual orientation (lesbian, gay, bisexual people), gender identity (trans people) and sex characteristics (intersex people). However significant obstacles remain to full recognition of LGBTI people’s fundamental rights. These rights include legal recognition of gender, non-discrimination in the workplace, freedom of expression and freedom of movement.^16^ Despite such advances however, social exclusion, stigmatization and discrimination experienced by LGBTI people persist in many healthcare settings.^17^^,^^18^ This is not only a social justice issue, but growing evidence links these experiences and related minority stress to health inequalities by showing that discriminatory behaviour can impact negatively on both mental health and physical health outcomes.^6^^,^^19^

As health inequalities have multiple root causes, reducing these inequalities is complex and there is no simple solution. Moreover, there is a significant lack of research regarding how to address these inequalities. Indeed, in 2016 this journal noted the need for greater international research to inform LGBT public health initiatives.^20^ Tackling inequalities requires a blended approach by addressing the fundamental causes of inequalities, preventing harmful wider social influences and mitigating against negative effects on individuals.^21^

Therefore, this global review was undertaken as part of an EU-funded pilot project that aimed to explore the sources of and modalities for reduction of LGBTI health and healthcare inequalities by determining: (i) what are the causes of LGBTI health inequalities? (ii) What is known about the health inequalities faced by LGBTI people as it relates to healthcare settings? (iii) What is known about the health inequalities of LGBTI people on vulnerable intersections (e.g. rural, younger, older, refugee, those in poverty or disabled)? (iv) What are the potential barriers faced by health professionals when providing care for LGBTI people and how can these barriers be addressed?

## Methods

A narrative synthesis design was used to search global literature systematically. This design was chosen due to the complex exploratory nature of the review which aimed to establish ‘what is known’ about LGBTI health and healthcare inequalities as well as produce a synthesis of current thinking that cuts across the field offering new perspectives and new areas for further research, training and policy development. Whilst such a review may not necessarily provide answers to addressing explicit health problems in given settings, it can nevertheless help policy makers, researchers and practitioners address concerns that occur across the data.^3^ In total, 57 relevant papers were extracted and reviewed including: systematic reviews (10), narrative reviews (3), reviews of systematic reviews (2), a meta-synthesis (1) and primary research (41).

### Search strategy

Systematic searches were carried out using six electronic databases [CINAHL, PsychINFO, MEDLINE (including PubMed), Web of Science, Cochrane Database of Systematic Reviews, Campbell Collaboration Library of Systematic Reviews]. Additional databases were excluded to prevent duplication. Google Scholar was searched in English and the references of included papers were then checked to identify further relevant articles.

### Key terms

Database searches were conducted using various combinations of key words and MeSH terms for the three main areas of interest: health inequalities, the study population (LGBTI people) and health professionals (healthcare inequalities or barriers to providing care for LGBTI people).

**Figure 1:**
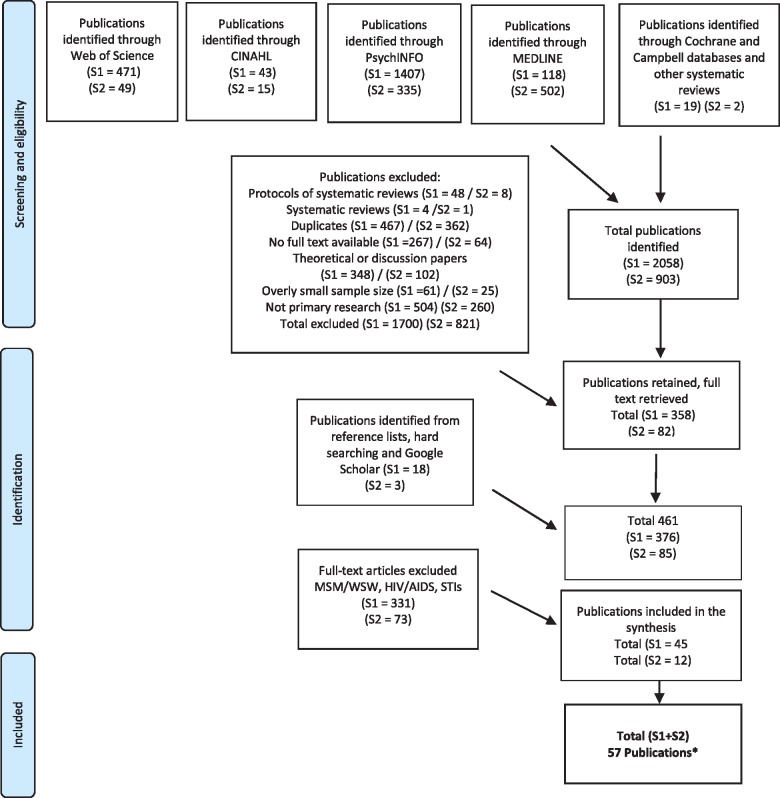
Selection procedure

Although some of the search terms used medicalize and or pathologize sexualities, gender identities and sex characteristics, these terms were included to ensure the broadest coverage and to expand retrieval. To maximize the number of relevant studies, literature searches were conducted in two parts (see figure 1) focussing on: search question one (S1) ‘health inequalities and the study population LGBTI people including vulnerable intersections such as rural, older, refugee, immigrant, disability, poverty’ and; search question two (S2) ‘the barriers health professionals encounter to providing care for LGBTI people’ (table 1).

**Table 1:** Key terms

Key search terms
**1) What is known about the health inequalities faced by LGBTI people as it relates to healthcare settings? (S1)** Lesbian / gay / homosexual^a^ / bisexual / trans^a^ / transgender / transsexual^a^ / intersex / hermaphroditism / disorders of sex development / queer / transvest^a^ / gender identity / questioning / unsure / LGBTI / GLBT / LGB / LGBT / LGBTQ / LGBTU / LGBT & I / same sex / same-sex / sexual minority / sexual orientation and / or Health inequality^a^/ disparity^a^ / gradient / disadvantage^a^ / determinant^a^ and / or
**What is known about the health inequalities of LGBTI people focussing on vulnerable intersections (e.g. rural, older, refugee, immigrant, disability, poverty) as it relates to healthcare? (S1)** Intersection^a^ / rural areas / rural population / rural health / aged / old^a^ / young / disab^a^/ poverty / migrants^a^ / immigrants/ asylum^a^/ refugee / displaced and / or
**What are the potential barriers faced by health professionals when providing care for LGBTI people? (S2)** Barrier^a^/ gap / beliefs / attitudes / values / norms / perspective / opinion / heteronormative^a^ / perception Health service accessibility / healthcare accessibility / health profession^a^ / staff / nurs^a^ / doctor / clinician^a^

a Journal requirements allow a maximum of 40 references. The full list and summary of 57 papers are thus provided in an accompanying Supplementary data.

### Selection criteria

Papers were considered for inclusion if they: (i) were primary research studies; (ii) reviews, systematic reviews or meta-analyses; (iii) were published from 2010 onwards to ensure the most recent studies were captured (except for the inclusion of two pivotal systematic reviews in the field published from 2008); and (iv) were published in English. All editorials, commentaries, non-research and theoretical papers were excluded.

### Data extraction

Eligibility for inclusion was assessed initially (by the first author) by screening all identified papers and reports based on titles and abstracts. The full text was then obtained for all selected articles and a second screening performed to determine final eligibility was agreed between the first and second author. Any discrepancies/disagreements were resolved in consultation with the third author. The data extraction process is summarized in figure 1. Geographical restrictions with Europe as a primary focus were applied with a wider international focus where relevant. Of the 57 papers included, 20 were European (any papers that included one or more EU countries), 37 were international (all other countries outside Europe which included America, Australia and Canada).

## Results

### Studies identified

The first database search on health inequalities and LGBTI people (identified as S1 in figure 1) extracted 2058 papers and 357 were selected for full-text review with 45 meeting the final inclusion criteria. The second database search on health professionals including barriers to providing culturally competent care for LGBTI people (identified as S2 in figure 1) identified 903 papers with 82 selected for full-text review and 12 meeting the final inclusion criteria. Combined, 57 papers were included in this review although only the 40 most relevant studies are cited here due to journal editorial restriction (for a full list of papers see the Supplementary data). Of the 57 papers, 16 were systematic reviews and/or meta-analyses and narrative reviews that each covered in the region of 25 research studies or more (16 systematic reviews × 25 papers each) meant more than 400 research studies were covered by this review. Moreover, papers that were published in addition to these systematic reviews or following these reviews, that met the inclusion/exclusion criteria, were also included. Due to the broad scope of the review, database searches were revisited several times to address gaps in the identified papers for specific (sub)populations e.g. the health outcomes of intersex people and their experiences of accessing healthcare. These iterative search measures were utilized to ensure each of the three questions were addressed in sufficient depth. Furthermore, the terms used to answer the review questions reflect the specific groups reported in research. Some papers reported on LGBT people, whereas others referred to LGB people or more specifically on trans or intersex people alone. These terms were honoured as they were presented in the original papers (table 2).

**Table 2:** Inclusion and exclusion criteria

Inclusion criteria (S1)	Exclusion criteria
Peer reviewed primary research articles published in academic journals, systematic reviews or narrative reviews	Grey literature
Large scope primary research	Overly small sample size
Published in English	Non-English
Published between 2010 and 2016	Prior to 2010
Social determinants	Biological and genetic factors
Physical and mental health	Sexual health
Homosexual, bi, trans and intersex	Sexual practices [e.g. WSW (women who have sex with women) and MSM (men who have sex with men) and sex work]^a^
Physical conditions including general health profile, cancer, weight discrepancies Mental conditions including suicide, depression, anxiety, mental distress, self-harm, substance misuse	HIV/AIDS and other STIs^b^
Rural, geographically remote areas	Urban areas
Over the age of 18 as per age of consent in EU MS^5^	Under the age of 18^c^
Older LGBTI people	LGBTI war veterans (USA)
Socioeconomic disadvantage or poverty	High income settings
Disabilities	
Migrants, immigrants, asylum seekers, refugees	
**Inclusion criteria (S2)**	**Exclusion criteria**
Acute care, community, hospitals, health promotion, surgeries, mental health services	Occupational health
Health professionals including gynaecologist, obstetrician, GP, psychologist, psychiatrist, mental health practitioners, nurse, midwife, surgeons, paediatrician, endocrinologist	Lay workers
Human care, treatment, practice	Animal care

a Research focussing on MSM and WSW were excluded as this review focussed on sexual orientation/identities instead of sexual practices.

b HIV/AIDS and other STIs were excluded due to being an already well-researched area and the resulting large and diverse literature available.

c Intersex research with participants under the age of 18 were included due to a peak in health service access during puberty and prior to the age of 18.

### What are the causes of LGBTI health inequalities?

In general, health inequalities occur due to the consequences of a complex interaction of social, cultural and political factors. For LGBTI people, the root causes likely to contribute to the experience of health inequalities are (i) cultural and social norms that preference and prioritize heterosexuality;^11^^,^^22^ (ii) minority stress associated with sexual orientation, gender identity and sex characteristics;^19^^,^^23^ (iii) victimization;^24^ (iv) discrimination (individual and institutional)^6^^,^^18^ and (v) stigma.^17^

Health inequalities occur in a context where heterosexuality prevails as the norm.^14^^,^^22^ LGBTI people access treatment and care in healthcare settings where it is often assumed that people are heterosexual, cisgender (not trans) and not intersex by default.^22^ These forms of heteronormativity and gender normativity can be understood as beliefs and practices where sex (male and female) and gender (masculinity and femininity) are absolute and unquestionable binaries. In heteronormativity opposite sex attraction or heterosexuality is the only conceivable way of being ‘normal’.^11^^,^^24^ As LGBTI people deviate from these norms insofar as their sexual orientation (LGB people), or gender identity (trans people), or sex characteristics (intersex people) they may experience discriminatory attitudes, prejudice or demeaning behaviour.^14^^,^^22^^,^^24^

Discrimination and prejudice sanction the behaviour of those who deviate from commonly accepted norms. The impact of discrimination is described in minority stress theory, the leading narrative explaining the health inequalities of LGBTI people.^12^^,^^19^^,^^23^ In brief, the minority stress model suggests that because of stigma, prejudice and discrimination, LGBTI people may experience more stress than non-LGBTI people, and that it is this disproportionate experience of stress that can lead to increased incidence of physical and mental health problems.^33^ Minority stress occurs where marginalized groups display specific risk factors. Whilst the entire population may display a particular risk factor, the incidence and effects of these risk factors may be more pronounced in smaller subsections of the larger population.^1^^,^^19^ Due to their minority status (e.g. LGB people only account for up to 6% of the UK population),^6^ LGB people were among the social groups most likely to experience higher levels of unpredictable, episodic and day-to-day social or minority stress because of discrimination and stigmatization,^17^^,^^19^ which creates a hostile environment where LGBTI people face stressful social exchange.^12^^,^^19^ A meta-analysis of 386 research studies with LGB people undertaken across 19 countries, reported up to 55% of people experienced verbal harassment, 45% experienced sexual harassment and 41% experienced discrimination at higher levels than the general population.^24^ For some LGBT people experiences of individual discrimination included hostility, personal rejection, harassment, bullying and violence,^18^ whilst for others institutional discrimination occurred where laws and policies in the public domain sustained inequalities such as the prohibition of same-sex marriage, or where laws did not protect against discrimination based on gender identity, sexual orientation or sex characteristics.^6^^,^^18^ Globally the degree to which LGBTI people are legally protected by anti-discrimination law and the level of legal and social recognition varied significantly. Where LGBTI people did not have legal protection, they were more apprehensive when accessing healthcare due to anticipated stigma;^12^^,^^17^ or LGBT people internalized stigma where they devalued themselves because of their gender identity or sexual orientation leading to significant barriers in accessing healthcare.^17^

### What is known about the health inequalities faced by LGBTI people?

Health inequalities were experienced differently between LGBTI groups and spanned both physical and mental health. LGB people reported significantly worse physical health compared to the general population with gay men showing an increased incidence of long-term conditions that restricted their activities of daily living. Conditions included musculoskeletal problems, arthritis, spinal problems and chronic fatigue syndrome,^6^ whereas gay and bisexual men showed a high incidence of long-term gastrointestinal problems, liver and kidney problems.^6^ Lesbian women had a higher rate of polycystic ovaries compared to women in general (80 vs. 32%)^6^ and both lesbian, gay and bisexual people showed weight discrepancies compared to the general population.^23^^,^^25^ Of LGB groups, the general health of bisexual people was poorer compared to lesbian and gay counterparts due to their minority status in both communities.^12^

LGB people are at a higher risk of developing certain types of cancer at a younger age.^26^ Gay and bisexual men are twice as likely to report a diagnosis of anal cancer with those who are HIV-positive being at the highest risk.^3^ Rates of anal cancer in gay and bisexual men are similar to the prevalence of cervical cancer in general female populations prior to the introduction of cervical screening programmes.^3^ This evidence supports the need for anal screening programmes geared towards gay and bisexual men. In contrast there was no conclusive evidence of higher rates of breast cancer in lesbian and bisexual women.^27^ However, LGB people who survived cancer reported the need for psychological and emotional support to address their specific needs.^28^ There is a gap in high quality international research on both the cancer burden, general health profile and care needs of trans and intersex people.^3^^,^^29^

In relation to mental health, significant inequalities exist with LGBT people being twice to three times more likely to report enduring psychological or emotional problems compared to the general population.^30^ Suicide attempts, suicidal ideation, depression and anxiety disorders were 1.5 times higher for LGB people compared to heterosexual peers with alcohol related substance dependence over the previous 12 months being 1.5 times more common in LGB people.^30^ Disparities related to mental distress were most pronounced for LGB people under the age of 35, and people over the age of 55.^1^ Intersex people also showed a raised incidence of suicide attempts at 19%, with 60% having considered suicide compared to 3% in mainstream populations.^29^ Bisexual and trans people showed even greater disparities in mental health compared to lesbian and gay counterparts, increasing the need for specialist mental health services and counselling support.^1^^,^^2^^,^^18^

Whilst accessing treatment and care, LGBTI people were more likely to report unfavourable experiences. General concerns were around communication with health professionals and overall dissatisfaction with treatment and care provided.^11^^,^^12^^,^^15^^,^^28^ Trans people frequently experienced negative interactions with health professionals at gender identity clinics, mental health services and general health services. Where trans people attended gender identity clinics, long waiting times for treatment was shown to negatively impact on their emotional wellbeing.^15^

Like LGBT people, some intersex people experience isolation due to stigma, discrimination or rejection from others.^29^ For some intersex people, experiences of adversity were linked to the medicalization of their bodies and being subjected to ‘normalising’ surgery at a young age or where their bodies were surgically aligned to male or female sex characteristics.^13^^,^^29^ Dissatisfaction about historic treatment was linked to health professionals not openly discussing information or failing to gain informed consent prior to surgical intervention on intersex minors.^5^

### What is known about the health inequalities of LGBTI people on vulnerable intersections?

In contemporary health and social care literature, it is well understood that there is a strong relationship between the social determinants of health inequalities and health outcomes.^10^ Various dimensions of social and cultural difference exist including gender, sexual orientation, gender identity, gender expression, sex characteristics, age, ethnicity, race, social class and disability among others.^12^ Intersectionality can be understood as the intersections between these dimensions associated with social and cultural difference, that people experience.^6^^,^^31^ People carry certain markers of difference and for LGBTI people these dimensions can intersect to create multiple marginalizations such as, young trans people experiencing high rates of mental distress where their gender, sexuality, and age intersect compounding the discrimination they face at school.^31^ Indeed, the literature shows that living in rural areas creates further health inequalities for LGBT people with reduced access to services, particularly for trans people.^17^ Older LGBTI people experienced both physical and mental health difficulties as they aged and became more dependent, however social support seemed to act as a protective factor.^32^^,^^34–40^ Conversely younger people appeared to be at risk of mental distress and substance misuse in ways that affected their educational attainment.^33^ However, targeted resources such as peer support were shown to have positive outcomes.^33^ LGBTI people on lower incomes were at risk of mental distress and were more likely to smoke, associated with other factors such as lack of social support and discrimination.^33^ LGBTI people were more likely to experience disabilities, and to be younger when doing so.^34^ LGBTI refugees and asylum seekers were likely to be at risk of physical and mental distress due to marginalization or abuse experienced in their country of origin linked to their sexual orientation, gender identity or sex characteristics,^35^ though further research is needed to understand fully and document the impact of intersectionality.

### What are the potential barriers faced by health professionals when providing care for LGBTI people and how can they be addressed?

Health professionals faced a range of challenges when caring for LGBTI people including heteronormativity where heterosexuality is upheld as the status quo or gender normativity where the male-female binary is retained as the norm.^22^^,^^24^ These norms were evident in practitioners own discomfort and unease whilst addressing the gender identity, sexual orientation or sex characteristics in conversations with LGBTI patients, combined with uncertainty about the use of language or terminology,^28^ and not knowing whether people were LGBTI or not.^36^ Health professionals were not always aware of key health needs of LGBTI people nor specific health conditions, and may unintentionally have been insensitive towards LGBTI people.^37^ Case notes and multidisciplinary forms often failed to recognize the lives and partnerships of LGBTI people.^14^ Relevant documentation like leaflets, marketing materials and processes for recording LGBTI patient information can help overcome barriers in communication where health professionals are encouraged to take account of gender and sexual diversity in clinical practice.^11^

When LGBTI people were recognized, or their lives and partnerships were acknowledged, they were more likely to be open and disclose their identity (‘come out’) or to share relevant health-related information.^11^ However some LGBT people had safety concerns or did not ‘come out’ due to their own need for privacy and confidentiality.^28^ Consequently health professionals may not have all the relevant information needed to make a full assessment or to suggest appropriate treatment options.^36^ Where LGBT people disclosed their gender identity or sexual orientation in health environments without negative consequences, their visibility correlated to a better rapport with health professionals.^17^

Further barriers occurred where health professionals lacked appropriate knowledge regarding the lives and related health needs of LGBTI people or where health professionals lacked the appropriate culturally specific skills necessary to meet their needs.^11^^,^^12^^,^^14^^,^^29^^,^^31^^,^^35^^,^^36^ As one of many examples, mixed methods research found only 41% of older LGBT people in healthcare thought health professionals had sufficient knowledge of LGBT issues whereas 59% thought health professionals did not have adequate knowledge.^36^ Global research reviewed was both clear and consistent in arguing for appropriate training of both specialist and generic health professionals to address key gaps in their knowledge and understanding when providing care,^31^^,^^35^^,^^36^^,^^38^ as well as informing LGBTI people of how to help reduce the barriers they face when accessing health services.^39^ With increased knowledge, health professionals working in partnership with LGBTI people, can contribute to reducing health inequalities.

## Discussion

This review has established ‘what is known’ about the health inequalities of LGBTI people and where change in practice or further research is needed. By identifying these gaps, the findings and recommendations can be of value for health policy makers, practitioners and researchers to help reduce these inequalities.

Recommendations stemming from this review include the need to address high rates of anal cancer in gay and bisexual men, by introducing anal screening programmes to ensure early detection.^3^ As for mental health, there were disparities in the mental distress of bisexual and trans people compared to gay and lesbian counterparts, resulting in the need for greater availability of specialist mental health services and counselling support for these groups.^1^^,^^2^^,^^18^^,^^39^ Specialist services are also required for intersex people with long-term follow-up and improved access to counselling support.^29^ The review showed lack of substantive research on the general health profile and cancer burden of trans and intersex people,^3^^,^^29^ with existing research often small in scale and limited in scope.^13^^,^^20^^,^^29^ Further large-scale research is needed to consider the general health and cancer burden of trans and intersex people and to explore their experiences of accessing healthcare. LGBTI people should be included in future research, policy initiatives and decisions about healthcare delivery to represent their own health concerns and to ensure their views of how to improve services are reflected.^6^^,^^11^^,^^31^

Very little research specifically considers how more than one factor intersect to influence the health outcomes of LGBTI people. Further research is needed to understand fully and document the potential impact of intersectionality. Where this kind of research did exist, studies showed that living in rural areas, being on a low income,^33^ being an LGBTI refugee or asylum seeker,^35^ being younger,^31^^,^^33^^,^^34^ or older^32^^,^^36^ and living with disabilities^34^ compounded the health inequalities of LGBTI people. Minority stress theory proposes that inequalities occur due to social, cultural and political factors where LGBTI people may experience discrimination associated with their minority status.^19^^,^^25^^,^^28^ In health settings where LGBTI people faced prejudice they were less likely to ‘come out’.^11^^,^^28^^,^^36^

Key but achievable changes are needed in healthcare to address the barriers that prevent access to care.^11^^,^^17^^,^^38^^,^^39^ This is essential action in line with European efforts to abolish discrimination on any grounds and to uphold and promote human rights.^7–9^^,^^16^ Recognition of LGBTI rights continue to vary significantly across European Member States.^16^ However structural change can be facilitated via policy, research and in practice combined with training of health professionals to improve their understanding of the lives, partnerships and health concerns of LGBTI people.^31^^,^^35^^,^^36^^,^^38^ Inclusion of LGBTI health and healthcare is imperative for curricula at universities and education centres where health professionals are trained. Health professionals will benefit from increased knowledge of historic events where ‘homosexuality’ was criminalized or medicalized as a ‘sexual disorder’, or where current framings of intersex variance as ‘disorders of sex development’ persist in systems of classification such as the WHO International Classification of Diseases (ICD-11) or the APA Diagnostic and Statistical Manual of Mental Disorders (DSM-V). An understanding of the marginalization of LGBTI people via these legal and medical frameworks may result in some avoiding disclosure in health settings acting as a barrier that prevents health professionals from providing effective care.^11^^,^^17^ Training should show how sustaining traditional heterosexual norms (heteronormativity) and binary gender (gender normativity) may be in tension with the equal rights afforded to LGBTI people in European Member States.^16^ With increased understanding of evolving diversity, practitioners can approach LGBTI people without judgement. Where health workers uphold professional values of inclusivity and respect in open communication,^31^^,^^35^^,^^36^^,^^38^ LGBTI people may be more empowered to disclose their specific health concerns during consultations.^11^^,^^17^ Health professionals could work in collaboration with LGBTI people towards a collective goal of truly inclusive and equally accessible services for all.

## Limitations

This review has made an important contribution to the field of health inequalities experienced by LGBTI people. Nevertheless, two key limitations should be noted. First, as a narrative synthesis, studies included were not assessed for quality and thus caution must be applied regarding interpretation and generalizability. Second, some of the studies reported in this review combined health profiles for lesbian and bisexual women, or gay and bisexual men or LGB people without considering the health inequalities of each individual group. In other words, our analysis revealed that studies commonly collapsed sexual minorities into a single group. Although combining data can be useful for analytical purposes, it may blur important issues specific to distinct groups and in some cases it was not possible to tease out such distinct issues. Future research designs should differentiate between LGBTI people to ensure analysis can be conducted separately without presuming their issues are the same in ways that neglect intersectional differences.

## Supplementary Material

cky226_Supplementary_Data
