# Supplementary material for: A review of lesbian, gay, bisexual, trans and intersex (LGBTI) health and healthcare inequalities
Source: Eur J Public Health. 2018 Oct 31;29(5):974–80. doi: 10.1093/eurpub/cky226 (PMC6761838; doi:10.1093/eurpub/cky226)
Supplement: cky226_Supplementary_Data [file cky226_supplementary_data.docx]

**Supplementary files**

Papers included in the review

| **Author** | **Title** | **Location** | **Method** | **Journal** |
| --- | --- | --- | --- | --- |
| 1. King *et al.* 2008 | A systematic review of mental disorder, suicide and deliberate self-harm in LGB people. | International and USA | Systematic review and meta-analysis | BMC Psychiatry |
| 1. Bauer *et al.* 2014 | Reported emergency department avoidance, use, and experiences of transgender persons in Ontario | Canada | Respondent-driven sampling (RDS) survey with n=433 trans people | Annals of Emergency Medicine |
| 1. Bauer *et al. 2015* | Intervenable factors associated with suicide risk in transgender persons: a respondent driven sampling study in Ontario, Canada. | Canada | RDS survey with N=380 trans people who reported on suicide outcomes | BMC Public Health |
| 1. Bailey *et al.* 2014 | Suicide risk in the UK trans population and the role of gender transition in decreasing suicidal ideation and suicidal attempt | UK | Survey with n=889 trans people | Mental Health Review Journal |
| 1. Blondeel *et al. 2016* | Evidence and knowledge gaps on the disease burden in sexual and gender minorities: a review of systematic reviews | Belgium and international | Narrative review of 30 systematic reviews | International Journal for Equity in Health |
| 1. Meads & Moore 2013 | Breast cancer in lesbians and bisexual women: systematic review of incidence, prevalence and risk studies | UK, USA and international | Systematic review | BMC Public Health |
| 1. Meads *et al. 2012* | Lesbian, gay and bisexual people's health in the UK: a theoretical critique and systematic review. | UK and international | Systematic review | Diversity and Equality in Health and Care |
| 1. Pennant *et al.* 2009 | Improving LGB healthcare. A systematic review of literature | UK and international | Systematic review | Diversity in Health and Care |
| 1. Katz-Wise & Hyde 2012 | Victimization experiences of lesbian, gay, and bisexual individuals: a meta-analysis. | USA and 18 countries | Meta-Analysis | Journal of Sex Research |
| 1. Goldbach *et al.* 2014 | Minority stress and substance use in sexual minority adolescents: A meta-analysis. | USA and international | Systematic review and meta-analysis | Prevention Science |
| 1. Elliott *et al.* 2015 | Sexual minorities in England have poorer health and worse healthcare experiences: A national survey | UK and USA | English General Practice patient survey of n=2,169,718 including 27,497 LGB | Journal of General Internal Medicine |
| 1. Eliason *et al.* 2015 | A systematic review of the literature on weight in sexual minority women | USA | Systematic review | Women’s Health Issues |
| 1. Sharek *et al.2015* | Older LGBT people's experiences concerns with healthcare professional and services in Ireland | Ireland | Survey and qualitative interviews with n=144 older LGBT people | International Journal of Older People Nursing |
| 1. Chakraborty *et al.* 2011 | Mental health of the non-heterosexual population of England | UK | Adult psychiatric morbidity survey n=7,403 | British Journal of Psychiatry |
| 1. Haas *et al. 2010* | Suicide and suicide risk in lesbian, gay, bisexual, and transgender populations: Review and recommendations | USA and international | Narrative review | Journal of Homosexuality |
| 1. Sanders *et al. 2015* | Young women with a disorder of sex development: learning to share information with health professionals, friends and intimate partners about bodily differences and infertility | UK | Interpretive phenomenological analysis with n=13 | Journal of Advanced Nursing |
| 1. Lee *et al.* 2012 | Review of recent outcome data of disorders of sex development (DSD): Emphasis on surgical and sexual outcomes | Sweden, USA, Belgium, Netherlands | Narrative review | Journal of Paediatric Urology |
| 1. Jones 2016 | The needs of students with intersex variations | Australia | Online survey with n=272 intersex participants | Sex Education |
| 1. Köhler et *al.* 2012 | Satisfaction with genital surgery and sexual life of adults with XY disorders of sex development: Results from the German clinical evaluation study | Germany, Austria, Switzerland | Psychosexual inquiry with n=57 intersex participants | Journal of Clinical Endocrinology & Metabolism |
| 1. Thyen *et al.* 2014 | Utilization of healthcare services satisfaction with care in adults affected by disorders of sex development (DSD) | Germany, Austria, Switzerland | Survey with n=110 intersex participants | Journal of General Internal Medicine |
| 1. Utamsingh *et al.* 2016 | Heteronormativity and practitioner–patient interaction | Durham, NC | LGBTQ and pansexual participants n=133, questionnaire, case study, written reflections | Health Communication |
| 1. Marques *et al.* 2015 | Lesbian medical encounters: Heteronormativity | Portugal | Individual interviews with n=30 lesbians | Healthcare of Women International |
| 1. Fish & Bewley 2010 | Using human rights-based approaches to conceptualise lesbian and bisexual women's health inequalities | UK | Questionnaire with LB women n=6,490 | Health and Social Care in the Community |
| 1. Bourne *et al.* 2016 | Physical health inequalities gay and bisexual men in England: A large community-based cross-sectional survey | UK | Online survey with gay and bisexual men n=5,799 | Journal of Public Health |
| 1. Blosnich *et al.* 2013 | A systematic review of tobacco inequalities for sexual minorities | USA and international | Systematic review | Tobacco Control |
| 1. Hickson *et al.* 2016 | Mental health inequalities among gay and bisexual men in England, Scotland and Wales: a large community-based cross-sectional survey | UK | Online survey with gay and bisexual men n=5,799 | Journal of Public Health |
| 1. Lyons A. *et al.* 2015 | Rural-urban differences in mental health, resilience, stigma and social support among young Australian gay men | Australia | Online survey with n=1,034 gay men | Journal of Rural Health |
| 1. Lyons, T. *et al.* 2015 | A qualitative study of transgender individuals’ experiences in residential addiction treatment settings: Stigma and inclusivity | Canada | Individual interviews with (n=34) trans people | Substance Abuse Treatment, Prevention, and Policy |
| 1. Whitehead *et al.* 2016 | Outness, stigma, and primary healthcare utilization among rural LGBT populations | USA | LGBT online survey n=1,014) | Plos One |
| 1. Alessi *et al.* 2016 | ‘The darkest times of my life’: Recollections of child abuse among forced migrants persecuted because of their sexual orientation and gender identity | USA & Canada | 26 individual interviews with refugee and asylum seekers | Child abuse and neglect |
| 1. Fish & Williamson 2016 | Exploring lesbian, gay and bisexual patients’ accounts of their experiences of cancer care in the UK | UK | 15 individual interviews LGB people | European Journal of CA care |
| 1. Boehmer *et al.* 2011a | Cancer survivorship and sexual orientation | USA | California health interview  Survey (n=122,394) | Cancer |
| 1. Boehmer *et al.* 2011b | An ecological analysis of colorectal cancer incidence and mortality: Differences by sexual orientation | USA | Census data (n=594,391) of same-sex partner households | BMC Cancer |
| 1. Boehmer *et al.* 2014 | Sexual minority population density and incidence of lung, colorectal and female breast cancer in California | USA | California cancer registry & national health interview survey | BMJ Open |
| 1. Hill & Holborn 2015 | Sexual minority experiences of cancer care: a systematic review | Scotland & international | Systematic review | Journal of Cancer Policy |
| 1. Cochran & Mays 2012 | Risk of breast cancer mortality among women cohabiting with same sex partners: Findings from the national health interview survey, 1997–2003 | USA | National health interview survey n=136,174 male and female n=692 | Journal of Women's Health |
| 1. Rose *et al.* 2016 | Let's talk about gay sex: Gay and bisexual men's sexual communication with healthcare professionals after prostate cancer | Australia | Survey n=124 and  46 interviews | European Journal of CA care |
| 1. Gonzales *et al.* 2016 | Comparison of health and health risk factors between lesbian, gay, and bisexual adults and heterosexual adults in the United States: results from the national health interview survey | USA | National health interview survey n=68,814 | JAMA Internal Medicine |
| 1. Gonzales & Henning-Smith 2015 | Inequalities in health and disability among older adults in same-sex cohabiting relationships | USA | National health interview survey n=698 men and n=630 women | Journal of Aging and Health |
| 1. Fredriksen-Golsen & Muraco 2010 | Aging and sexual orientation: A 25-Year Review of the Literature | USA | Narrative review | Research on Aging |
| 1. Fredriksen-Goldsen *et al*. 2013 | Health inequalities among lesbian, gay, and bisexual older adults: Results from a population-based study | USA | Survey data from the Behavioural risk factor surveillance system n=96 992 | American Journal of Public Health |
| 1. Fredriksen-Golsen *et al.* 2012a | The physical and mental health of lesbian, gay male, and bisexual (LGB) older adults: the role of key health indicators and risk and protective factors | USA | Survey with n=2,439 older adults, phone interviews | The Gerontologist |
| 1. Fredriksen-Golsen *et al*. 2012b | Disability among lesbian, gay, and bisexual adults: Disparities in prevalence and risk | USA | Survey data from the Behavioural risk factor surveillance system N=96 992 (n=82,531) | American Journal of Public Health |
| 1. Fredriksen-Golsen *et al*. 2010 | Disparities in health-related quality of life: A comparison of lesbians and bisexual women | USA | Survey data from the Behavioural risk factor surveillance system n=1,496) | American Journal of Public Health |
| 1. Wao *et al.* 2016 | MSM's versus healthcare providers’ perceptions of barriers to uptake of interventions: Systematic review, meta-synthesis | USA | Systematic review and meta-synthesis | International Journal of Sexual Health |
| 1. Zeeman *et al.* 2016 | Promoting resilience and emotional well-being of transgender young people: research at the intersections of gender and sexuality | UK | Participatory qualitative methods including focus group with trans youth (n=5) | Journal of Youth Studies |
| 1. Moe & Sparkman 2015 | Assessing service providers at LGBTQ-affirming community agencies on their perceptions of training needs and barriers to service | USA | Survey of service providers (n=109) | Journal of Gay & Lesbian Social Services |
| 1. Semlyen *et al.* 2016 | Sexual orientation and symptoms of common mental disorder or low wellbeing: combined meta-analysis of 12 UK population health surveys | UK | Meta-synthesis | BMC Psychiatry |
| 1. Reisner *et al.* 2014 | Transgender health inequalities: comparing full cohort and nested matched-pair study designs in a community health centre | USA | Health surveillance survey (n=155) | LGBT Health |
| 1. Reisner *et al.* 2016 | Global health burden and needs of transgender populations: a review | 30 countries including EU | Review and synthesis | Lancet |
| 1. Van Beusekom *et al.* 2016 | Gender nonconformity and mental health among lesbian, gay, and bisexual adults: Homophobic stigmatization and internalized homophobia as mediators | The Netherlands | Online survey (n=748) with LGB individuals | Journal of Health Psychology |
| 1. Maguen & Shipherd 2010 | Suicide risk among transgender individuals | USA | A survey with trans participants (n=153) to assess suicidal risk | Psychology & Sexuality |
| 1. Nokoff *et al.* In press | Prospective assessment of cosmesis before and after genital surgery. | USA | Prospective observational study with children <2 years of age (n=37) | Journal of Pediatric Urology |
| 1. Sherriff *et al.* 2011 | "What do you say to them?" Investigating and supporting the needs of lesbian, gay,  bisexual, trans, and questioning (LGBTQ) young people | UK | Qualitative methods with 29 young people and practitioners | Journal of Community Psychology |
| 1. Ellis *et al.* 2015 | Trans people’s experiences of mental health  and gender identity services: A UK study | UK | Survey of trans people n=889) | Journal of Gay & Lesbian Mental Health |
| 1. Budge *et al.* 2013 | Anxiety and depression in transgender individuals: The roles of transition status, loss, social support, and coping | USA | Various online measures with trans participants (n=351) | Journal of Consulting and Clinical Psychology |
| 1. Colledge *et al.* 2015 | Poorer mental health in UK bisexual women than lesbians: evidence from the UK 2007 Stonewall Women’s Health Survey | UK | Survey with n=937 bisexual-identified and n=4,769 lesbian-identified women | Journal of Public Health |
